# Molecular docking analysis of natural compounds as TNF-α inhibitors for Crohn's disease management

**DOI:** 10.6026/97320630019716

**Published:** 2023-06-30

**Authors:** Mamdoh S Moawadh

**Affiliations:** Medical Technology Department, Faculty of Applied Medical Sciences, University of Tabuk, Saudi Arabia

**Keywords:** Crohn's disease, TNF-α, natural compound, drug-likeness

## Abstract

Crohn's disease (CD) is a type of inflammatory bowel disease that is immune-mediated and affects the gastrointestinal tract. The chronic and severe nature of this condition leads to diminished health-related life quality, and frequent hospitalization. While medications such as sulfasalazine, corticosteroids, and immuno-suppressants are used to manage the condition, there are no definite treatments for pain and inflammation associated with CD. TNF-α is a prominent target, and medicines such as infliximab and adalimumab have pharmacological efficacy; however, they also have significant toxicity. Here, the natural compound library (2706 compounds) was screened against TNF-α to find natural TNF-α inhibitors to combat CD. The compounds namely ZINC5223934, ZINC6482465, ZINC4098633, ZINC1702729, and ZINC4649679 had higher binding affinity and interaction with the TNF-α protein than the positive control. Furthermore, these compounds had promising drug-like properties, indicating their potential for future exploration and optimization as TNF-α inhibitors for the treatment of CD.

## Background:

Crohn's disease (CD) is an inflammatory bowel disease (IBD) that is immune-mediated and affects the GI tract. It is a chronic condition with periods of remission and relapse. The disease's symptoms include recurrent diarrhea, stomach pain, and presence of blood in the stools. The combination of disease severity and chronicity results in reduced health-related life quality, and frequent hospitalization [1]. Tumor necrosis factor (TNF) is a molecular mediator that plays a vital role in the onset and progression of intestinal inflammation, which is a hallmark of CD [2]. TNF-α is a multifunctional proinflammatory cytokine that participates in diverse cellular mechanisms such as cell proliferation, survival, and apoptosis. Furthermore, TNF-α signaling is intricately linked to the modulation of different inflammatory pathways, particularly involving the COX-2 and iNOS pathways [3, 4, 5]. Therefore, TNF-α emerges as a pivotal mediator in the inflammatory response. The goal of anti-TNF-α medication is to counteract the actions of TNF-α. In the clinical management of IBD, a range of anti-TNF-α medications, such as infliximab, adalimumaab, golimumab, certolizumab, etanercept, and onercept have been used or are currently being used [6]. Computer-assisted drug design (CADD) has emerged as a viable technique for identifying promising leads and expediting the development of new medications to treat a wide range of ailments [7, 8]. A variety of computational tools are now being used to identify leads from huge chemical libraries. The use of CADD in drug development is fast progressing, with a rising emphasis on the rational design of effective treatments with multi-targeting capabilities, enhanced efficacy, and fewer side effects, particularly in respect to toxicity issues [9]. This study aimed to find the natural compound TNF-α inhibitor to fight the CD.

## Material and Methods:

## Protein preparation:

The crystal structure of TNF-α (PDB ID: 2AZ5) was obtained from PDB [10]. Protein was cleaned by removing heteroatoms including inbound ligand and water atoms. Loop modelling was performed to complete the distorted structure and then protein was prepared for subsequent screening of natural compounds library, which included assigning bond ordering using the "Protein Preparation Wizard" tool and conducting a restricted energy minimization using Discovery Studio 2021.

## Natural compound library preparation:

A compound library of 2706 natural compounds from the ZINC database was retrieved in sdf format and all the compounds were subjected to energy minimization using 'uff' force field and prepared for further screening.

## Docking based virtual screening:

Virtual screening is a valuable technique employed to expand databases by identifying active compounds and filtering out inactive ones before their experimental validation in the laboratory. Computational methods are utilized to analyze extensive datasets comprising known 3D structures [11]. Here, the PyRx 0.8 tool [12] was utilized to screen the prepared compound library against the TNF-α active site. The TNF-α grid center coordinates were set as X = −8.333, Y = 68.217, and Z = 19.962. Subsequent to the screening, a thorough interaction analysis was conducted, with a focus on compounds exhibiting lower binding energy values, in order to identify the most stable complex.

## Physicochemical properties and toxicity endpoints prediction:

The SwissADME web server [13] and the ProTox-II [14] platform were utilized to evaluate the physicochemical properties and predictions of different toxicity endpoints for five selected compounds. The ProTox-II server computed various toxicity parameters, including acute toxicity, hepatotoxicity, cytotoxicity, carcinogenicity, mutagenicity, immunotoxicity, and toxicity targets.

## Results and Discussion:

TNF-α plays a crucial role as a central mediator in the inflammatory response, and it is widely recognized that CD is an IBD. The goal of this study was to target the TNF-α protein and identify natural compounds that could act as potential inhibitors. This approach was chosen because previously reported inhibitors demonstrated beneficial effects while also exhibiting toxicity issues. Consequently, the investigation of natural compound-based therapy was deemed necessary in order to avoid toxicity concerns and potential adverse effects. Here, the computational methodology included the virtual screening of natural compound library (2706 compounds) retrieved from the ZINC database against the TNF-α active site pocket. The co-crystal inhibitor of the PDB (2AZ5) (6,7-dimethyl-3-[(methyl{2-[methyl({1-[3-(trifluoromethyl) phenyl]-1h-indol-3-}methyl)amino]ethyl}amino) methyl]-4h-chromen -4-one) (SPD304) was selected as positive control. Several compounds have been identified to bind with the TNF-α active site residue with comparable binding energy to the control. Table 1 describes the top 10 compounds in terms of binding energy and other physicochemical properties. The physicochemical properties including topological polar surface area (TPSA), MolLogP, H-bond acceptors and donors, number of rotatable bonds etc. determines the compound's ability of interaction with active site of the target proteins.

**Figure 1:**
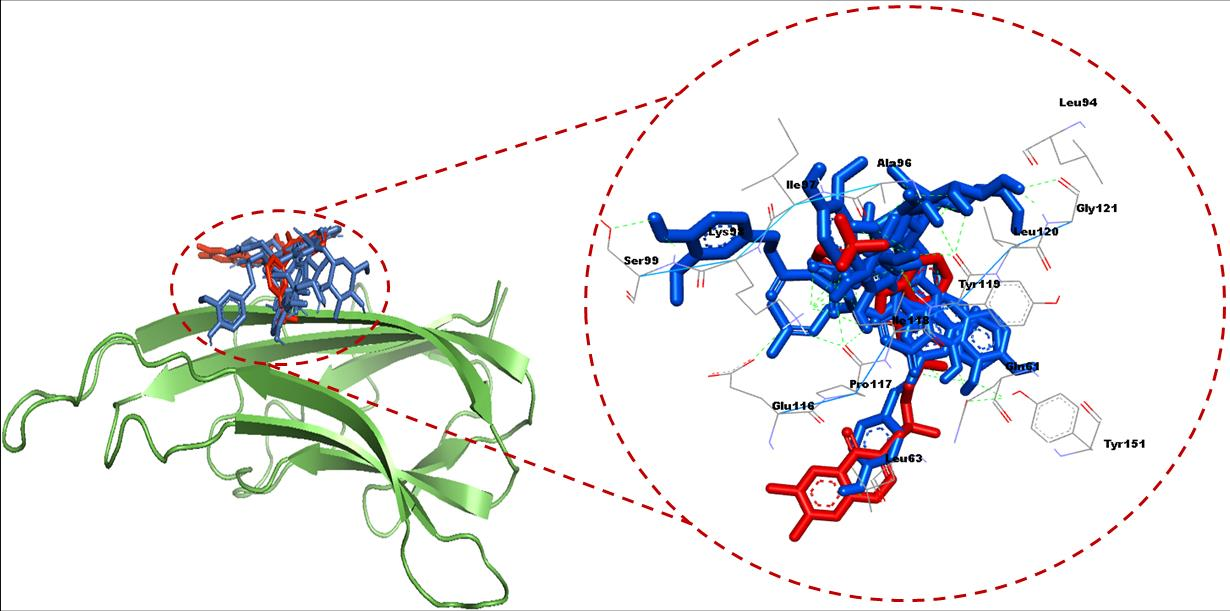
Visualization of top 5 compounds and positive control in TNF-α active site.

**Figure 2:**
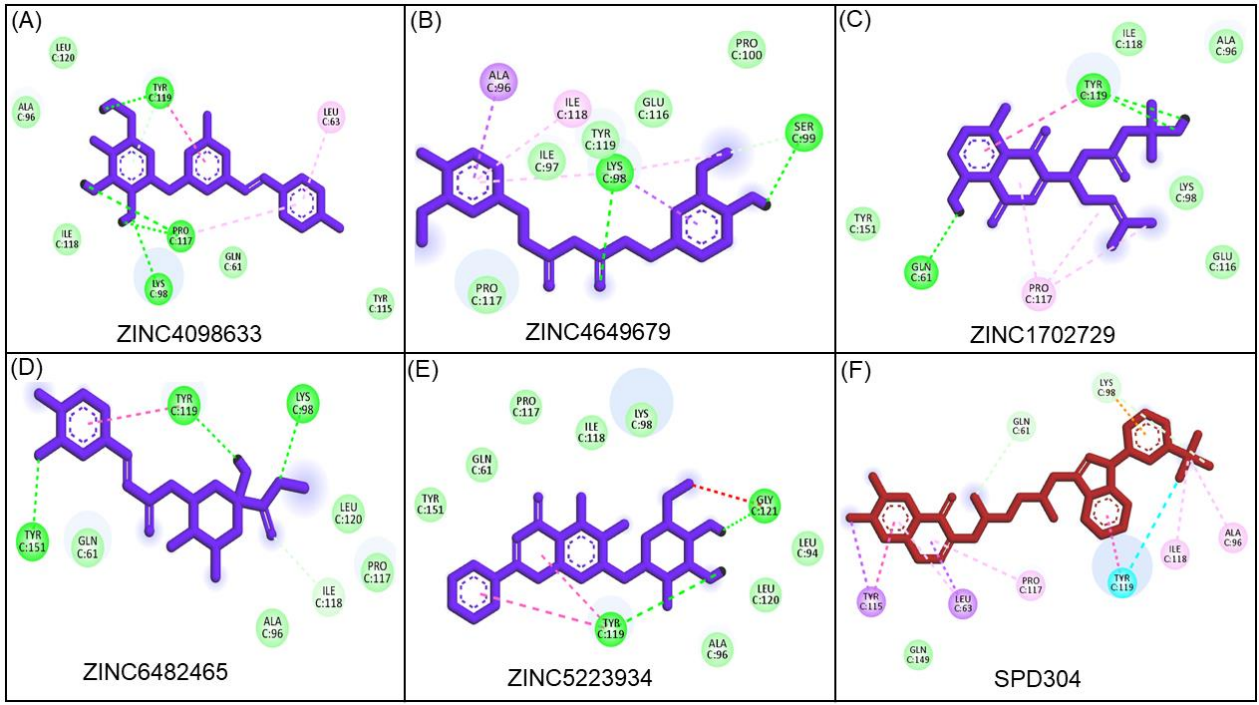
Interacting residues of TNF-α protein with top 5 compounds and positive control.

Further, by considering the compounds properties and visualization of the docked poses, a comprehensive analysis of the specific interactions was conducted for the five most promising hit compounds namely ZINC5223934, ZINC6482465, ZINC4098633, ZINC1702729, and ZINC4649679 (Figure 1). ZINC4098633 interacted with Ala96, Leu120, Tyr119, Ile118, Pro117, Cys98, Gln61, Leu63, and Tyr115 residues of TNF-α protein. Lys98, Pro117, and Tyr119 residues were H-bonded, while Ala96, Leu120 Ile118, Gln61, and Tyr115 residues were involved in van der waals interactions with ZINC4098633 (Figure 2A). ZINC4649679 interacted with Ala96, Ile118, Ile97, Tyr119, Lys98, Glu116, Pro100, Ser99, and Pro117 residues of TNF-α protein. Lys98, and Ser99 residues were H-bonded with ZINC4649679 (Figure 2B). ZINC1702729 interacted with Tyr151, Gln61, Tyr119, Ile118, Ala96, Lys98, Glu116, and Pro117 residues of TNF-α protein. Gln61 and Tyr119 residues were H-bonded with ZINC1702729 (Figure 2C). ZINC6482465 interacted with Tyr151, Gln61, Tyr119, Lys98, Leu120, Pro117, Ile118, and Ala96 residues of TNF-α protein. Tyr151, Tyr119, and Lys98 residues were H-bonded with ZINC6482465 (Figure 2D). Further, ZINC5223934 interacted with Tyr151, Gln61, Pro117, Ile118, Lys98, Gly121, Leu94, Leu120, Ala96, and Tyr119 residues of TNF-α protein. Tyr119 and Gly121 residues were H-bonded with ZINC5223934 (Figure 2E). SPD304 is a TNF-α inhibitor that increases the dissociation of TNF trimers, hence blocking the interaction between TNF and its receptor [10]. TNF-α protein residues that interact with its co-crystallized inhibitor SPD304 were investigated by re-docking SPD304 with TNF-α, which revealed that Tyr115, Gln149, Leu63, Pro117, Gln61, Lys98, Ala96, Ile118, and Tyr119 TNF-α residues were involved in SPD304 binding (Figure 2F). Notably, the hit compounds were found to bind with these TNF-α residues, with Pro117, Ala96, Ile118, and Tyr119 being the common binding residues with hit compounds (ZINC4098633, ZINC4649679, ZINC1702729, ZINC6482465, and ZINC5223934) and SPD304 (Figure 2A-2F).

**Figure 3:**
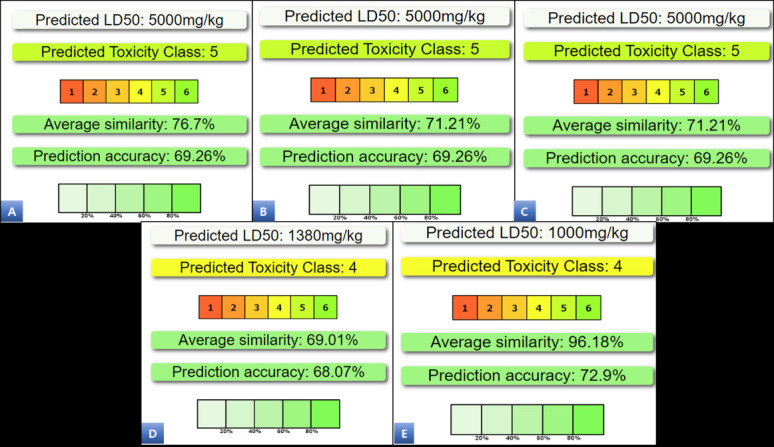
Oral toxicity prediction of ZINC5223934 (A), ZINC6482465 (B), ZINC4098633 (C), ZINC1702729 (D), and ZINC4649679 (E)

The minimal binding energy acquired from docking explains the ligand binding efficiency with the target protein [15,16]. Notably, the hits (ZINC5223934, ZINC6482465, ZINC4098633, ZINC1702729, and ZINC4649679) exhibit considerably lower binding energies than the SPD304 (control), indicating a strong interaction between these hits and the TNF-α protein. The prediction of compound toxicities plays a critical role in the drug design and development process. To address this, ProTox-II integrates several methodologies, including molecular similarity, fragment propensities, frequent feature analysis, and machine learning-based models. These approaches are employed to predict a wide range of toxicity endpoints. By leveraging these diverse techniques, ProTox-II provides comprehensive insights into the potential toxic effects of compounds, aiding in the identification of safer and more effective drug candidates. The predicted values for the five selected compounds indicate that they fall within an acceptable range for toxicity assessment (Figure 3 and Table 2).

##  Conclusion:

This study presents the virtual screening approach for identifying natural compounds that can target the TNF-α active site. The compounds namely ZINC5223934, ZINC6482465, ZINC4098633, ZINC1702729, and ZINC4649679 demonstrated strong binding affinity and interaction with the TNF-α protein. Furthermore, these compounds displayed favorable characteristics in terms of their drug-like properties, indicating their potential for further investigation and optimization as TNF-α inhibitors for the management of CD.

## Figures and Tables

**Table 1:** Top 10 screened compounds with binding energy and physicochemical properties.

**Compounds**	**Binding energy (kcal/mol)**	**MolWt**	**MolLogP**	**H_Acceptors**	**H_Donors**	**Rotatable Bonds**	**TPSA**
ZINC5223934	-7.4	432.381	0.0499	10	6	4	170.05
ZINC6482465	-7.2	368.338	-0.5575	9	5	4	153.75
ZINC4098633	-7.1	368.338	-0.5575	9	5	4	153.75
ZINC1702729	-6.9	390.388	0.4469	8	6	5	139.84
ZINC4649679	-6.9	388.416	2.8323	7	3	6	121.13
ZINC2585546	-6.8	278.307	3.29102	3	0	0	43.37
ZINC3978504	-6.8	716.604	3.1949	16	11	4	284.36
ZINC4098521	-6.8	538.464	5.5537	10	5	4	170.8
ZINC895645	-6.7	333.343	3.914	5	0	0	40.16
ZINC706	-6.8	608.549	-1.0897	15	8	7	238.2
SPD304*	-6.5	-	-	-	-	-	-
*Positive control

**Table 2:** Toxicity Model Computation of top 5 compounds

**Classification**	**Target**	**ZINC5223934**	**ZINC6482465**	**ZINC4098633**	**ZINC1702729**	**ZINC4649679**
**Prediction**	**Probability**	**Prediction**	**Probability**	**Prediction**	**Probability**	**Prediction**	**Probability**	**Prediction**	**Probability**
Organ toxicity	Hepatotoxicity	X	0.82	X	0.74	X	0.74	X	0.85	X	0.65
Toxicity end points	Carcinogenicity	X	0.85	X	0.71	X	0.71	X	0.81	X	0.57
Immunotoxicity	X	0.92	Active	0.99	X	0.99	Active	0.74	Active	0.87
Mutagenicity	X	0.76	X	0.86	X	0.86	X	0.73	X	0.69
Cytotoxicity	X	0.69	X	0.8	X	0.8	X	0.85	X	0.6
Tox21-Nuclear receptor signaling pathways	Aryl hydrocarbon Receptor	X	0.92	X	0.95	X	0.95	X	0.81	X	0.83
Androgen Receptor	X	0.9	X	0.95	X	0.95	X	0.89	X	0.94
Androgen Receptor Ligand Binding Domain	X	0.98	X	0.95	X	0.95	X	0.95	X	0.84
Aromatase	X	1	X	0.92	X	0.92	X	0.96	X	0.8
Estrogen Receptor Alpha	X	0.91	X	0.85	X	0.85	X	0.76	X	0.72
Estrogen Receptor Ligand Binding Domain	X	0.99	X	0.91	X	0.91	X	0.94	X	0.86
PPAR-Gamma	X	0.99	X	0.96	X	0.96	X	0.98	X	0.92
Tox21-Stress response pathways	nrf2/ARE	X	0.98	X	0.92	X	0.92	X	0.95	X	0.7
Heat shock factor response element	Inactive	0.98	X	0.92	X	0.92	X	0.95	X	0.7
Mitochondrial Membrane Potential	X	0.98	X	0.75	X	0.75	X	0.84	X	0.52
Phosphoprotein (Tumor Supressor) p53	Active	0.5	X	0.82	X	0.82	X	0.71	X	0.56
ATPase family AAA domain-containing protein 5	X	1	X	0.93	X	0.93	X	0.93	X	0.87
